# COVID-19 Point-of-Care Diagnostics That Satisfy Global Target Product Profiles

**DOI:** 10.3390/diagnostics11010115

**Published:** 2021-01-12

**Authors:** Abdi Ghaffari, Robyn Meurant, Ali Ardakani

**Affiliations:** 1 Department of Pathology and Molecular Medicine, Queen’s University, Kingston, ON K7L 3N6, Canada; 2 Novateur Ventures Inc., Vancouver, BC V6E 3P3, Canada; 3 NSF Health Sciences, Kirkbymoorside, York YO62 6AF, UK; rmeurant@nsf.org

**Keywords:** COVID-19, point-of-care diagnostic test, target product profile, clinical performance

## Abstract

COVID-19 pandemic will continue to pose a major public health threat until vaccination-mediated herd immunity is achieved. Most projections predict vaccines will reach a large subset of the population late in 2021 or early 2022. In the meantime, countries are exploring options to remove strict lockdown measures and allow society and the economy to return to normal function. One possibility is to expand on existing COVID-19 testing strategies by including large-scale rapid point-of-care diagnostic tests (POCTs). Currently, there is significant variability in performance and features of available POCTs, making selection and procurement of an appropriate test for specific use case difficult. In this review, we have used the World Health Organization’s (WHO) recently published target product profiles (TPPs) for specific use cases of COVID-19 diagnostic tests to screen for top-performing POCTs on the market. Several POCTs, based on clinical sensitivity/specificity, the limit of detection, and time to results, which meet WHO TPP criteria for direct detection of SARS-CoV-2 (acute infection) or indirect diagnosis of past infection (host antibodies), are highlighted here.

## 1. Introduction

Despite recent successes in vaccine development, the COVID-19 pandemic will continue to pose a major public health threat until a significant number of the global population is vaccinated and herd immunity is achieved. In the meantime, countries are exploring options to balance between preventing the further spread of SARS-CoV-2 and softening the societal lockdown that has caused major political and financial crisis. Most projections predict reaching herd immunity to SARS-CoV-2, primarily by mass vaccination [1], in the fourth quarter of 2021 [2]. A proposed solution for ending the lockdown is the large-scale utilization of rapid point-of-care diagnostic tests (POCTs) into the current COVID-19 testing, tracking, and tracing strategy. Such strategies can help mitigate the impact of the pandemic on vulnerable populations while allowing for society and the economy to continue to function [3,4].

The current gold standard for the diagnosis of acute SARS-CoV-2 infection is the reverse transcription polymerase chain reaction (RT-PCR) test that can detect small amounts of viral nucleic acid (SARS-CoV-2 RNA) in clinical specimens (e.g., nasopharyngeal swabs) with high accuracy [5,6]. However, RT-PCR usually requires expensive equipment and reagents that have limited its application to centralized laboratories with highly trained laboratory personnel, and typically a turnaround time of one to several days from specimen collection to the issuance of a result. The management of COVID-19 infection can be severely hindered by such long turnaround times [4]. Furthermore, expanding laboratory-based PCR testing capacity is beyond the financial means of many low- and middle-income countries and its logistics make it less agile to use as a near-patient or community-based test.

**Figure 1:**
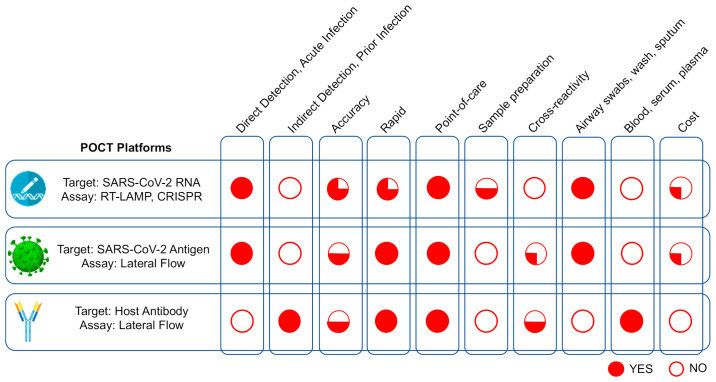
Features of various COVID-19 point-of-care diagnostic test (POCT) technology platforms. RT-PCR, reverse transcription polymerase chain reaction; CRISPR, cluster regularly interspaced short palindromic repeats; RT-LAMP, reverse transcription loop-mediated isothermal amplification. Adapted and modified from Ghaffari et al., BioProcess International, 2020 [2].

POCTs or near-patient tests are rapid decentralized (outside centralized laboratories) tests that can diagnose acute or prior SARS-CoV-2 infection within minutes of specimen receipt, allowing for rapid decisions concerning patient care and management to prevent further spread (see Box 1). POCTs can be divided into tests that directly detect SARS-CoV-2 (RNA or antigen) for acute diagnosis of COVID-19, or indirectly, by detecting host anti-SARS-CoV-2 antibodies for diagnosis of prior infection [3] (Figure 1). Direct POCTs that detect viral RNA or antigen(s) are available in several formats which are suitable for decentralized testing. Other than RT-PCR, these include lateral flow tests for antigen detection, RT-LAMP (reverse transcription loop-mediated isothermal amplification), and CRISPR (clustered regularly interspaced short palindromic repeats) for RNA detection. Indirect POCTs that detect antibodies have primarily relied on a lateral flow assay format to detect host antibodies (IgG, IgM, and IgA) from a small volume of blood, serum, or plasma [6]. Compared with RT-PCR, direct POCTs generally have lower sensitivity and can potentially detect SARS-CoV-2 during the first week after the onset of symptoms while the viral load is typically high. Beyond 10 to 14 days after the onset of symptoms, when the viral load is low or undetectable, the performance of these tests diminishes significantly [3,7]. Although of limited use in diagnosing recent infection, COVID-19 antibody-based POCT can be used to identify prior infection or effective vaccination by detecting host antibodies produced against SARS-CoV-2 antigens, which normally peak after 10 days post onset of symptoms [3,8].

It is important to highlight that significant variability in the SARS-CoV-2 incubation periods and kinetics, host immune and antibody response to the virus, and COVID-19 clinical phenotypes among individuals can limit the effectiveness of diagnostic tests. In addition, the performance of tests can be influenced by several confounding factors such as disease severity and stage, patient age, sampling site and collection method, virus strain subtype, presence of other respiratory pathogens, and technical errors which can lead to false negative or false positive results (limitations of COVID-19 tests have been discussed elsewhere [3,7,9,10,11,12]). As a result, a one-size-fits-all approach to the use of COVID-19 POCT is not feasible in response to the SARS-CoV-2 pandemic. Hence, target product profiles (TPPs) have been developed for specific use cases (such as for diagnosis, confirmation, or for surveillance) and target populations to guide industry efforts and help countries to define their testing strategies.

According to the World Health Organization (WHO), a TPP outlines the desired profile or characteristics of a target product that is aimed at a particular disease or diseases [13]. The TPP identifies specific design attributes addressing safety and performance that would be desirable in tests for the specific use cases. Thus, for a manufacturer, a TPP can be an invaluable strategic planning tool, providing critical input into the design process and clarity on the goals and expectations for the development of a needed diagnostic test. Generally, a TPP does not include information that would guide a manufacturer as to how to achieve these characteristics and what validation and verification activities are expected to be performed. For instance, the performance level in terms of clinical sensitivity and specificity that are needed to support a COVID-19 diagnostic test are described in a TPP, usually without reference to the fact that this should be attained for each major variant of SARS-CoV-2 circulating in the population. For countries, TPPs can be used to help identify potential candidate devices that can support the response to the pandemic for a specific intended use case. As the pandemic surged, many COVID-19 diagnostic tests were rapidly available on the market. Not all proved to meet their performance claims in various use case settings, as many had been designed without consideration of the unique viral and humoral kinetics, and the specific needs of different use cases. Currently, there are more than 300 COVID-19 diagnostic tests that have obtained U.S. Food and Drug Administration (FDA) Emergency Use Authorization (EUA) [14] and over 850 tests listed on the Swiss-based Foundation for Innovative New Diagnostics (FIND) [15]. Navigating hundreds of diagnostic tests to select the most reliable and accurate POCTs for procurement decisions has proved extremely challenging. In this review, we have screened the FDA EUA and FIND databases to select top-performing POCTs that meet WHO TPP criteria for direct (RNA/antigen) or indirect (antibody) COVID-19 diagnostic tests [13] for the use cases of detection of current and past infection.

Box 1 **Benefits and challenges of POCTs.**

*Definitions:*

▪ *Rapid Test*: a qualitative or semi-quantitative in vitro diagnostic medical device, intended to be used singly or in a small series, which involves non-automated procedures and has been designed to give a fast result.
▪ *Point of Care Testing*: testing that is performed near or at the site of the patient, outside a general laboratory environment, with the result leading to possible change in the care of the patient.

*Potential advantages:*

▪ Improved turnaround time
▪ Improved monitoring during pandemics where frequent testing is desirable
▪ Smaller sample (may be less invasive) and reagent volumes
▪ Advantages in remote regions where access to laboratory is limited
▪ Economic—POCTs may offer wider economic benefit with a reduced number of clinical visit and fewer hospital admissions
▪ Greater patient involvement in their own care, improved patient experience
▪ Availability outside core laboratory normal hours

*Potential disadvantages:*

▪ Reduced quality of analysis (e.g., sensitivity/specificity)
▪ Poor record keeping
▪ Lack of result interpretation
▪ Unnecessary duplication of equipment
▪ Data recording may be complex and less robust
▪ Incompatibility with laboratory results
▪ POCT can be expensive in absence of economies of scale that come from centralized laboratory testing

## 2. Target Product Profiles of COVID-19 Rapid Diagnostic Tests

In addition to key parameters that measure the analytical and clinical performance of a diagnostic test (see Box 2), other practical and strategic criteria play a significant role in the selection of a POCT for a specific use case. The WHO has recently called for research and development of simple, rapid, and more affordable COVID-19 POCTs and also encouraged the use of serological or antibody surveys to better understand the extent of and risk factors of this pandemic. To guide these efforts, the WHO and other jurisdictions such as the UK Medicine and Healthcare products Regulatory Agency (MHRA) have published four priority, target product profiles (TPPs), for the following use cases [13,16,17]:

1. Point-of-care test for suspected COVID-19 cases and their close contacts to diagnose acute SARS-CoV-2 infection;
2. Test for diagnosis or confirmation of acute or subacute SARS-CoV-2 infection, suitable for high-volume needs;
3. Point-of-care test for prior infection with SARS-CoV-2;
4. Test for prior infection with SARS-CoV-2 for high volume needs.

Box 2 **Performance parameters and variability of POCT [1].**

*Analytical Sensitivity*: or limit of detection (LOD) is frequently defined as the lowest amount of analyte (e.g., SARS-CoV-2 RNA) that can be accurately measured by the assay. The LOD of a COVID-19 POCTs is typically determined as the lowest SARS-CoV-2 analyte concentration (titrated at increasing concentration into a background) that was detected ≥95% of the time (at least 19 out of 20 replicates tested positive).

*Analytical Specificity*: or cross reactivity is the ability to unequivocally detect a specific analyte (e.g., SARS-CoV-2) and differentiate it from other interfering substances (e.g., other pathogens). The cross-reactivity and potential microbial interference of a COVID-19 test is typically evaluated by testing a panel of commensal and respiratory pathogenic microorganisms (bacteria, viruses, yeast) in the absence or presence of heat inactivated SARS-CoV-2 virus.

*Clinical Sensitivity and Specificity*: clinical or diagnostic sensitivity is the ability of a test to return a positive result when the patient has the disease. Clinical specificity is the ability of the test to produce a negative result when the patient sample does not have the disease. The clinical performance of a COVID-19 POCT is evaluated by using confirmed positive and negative SARS-CoV02 clinical specimen. Positive SARS-CoV-2 specimen are typically from patients who presented within 7 days of COVID-19 symptom onset.

*Clinical specimen*: for antigen tests, the quality and relevant abundance of SARS-CoV-2 in collected clinical specimens, heavily dependent on the collection site and disease timeline, are critical for the performance evaluation of the assays. For example, the sensitivity of RT-PCR in detection of SARS-CoV-2-infected patients is ~93% in bronchoalveolar lavage fluid, 63% in nasopharyngeal swabs, 72% in sputum, 32% in pharyngeal swabs, and 29% in stool [5]. Furthermore, at ~14 days after the onset of COVID-19 symptoms the viral load becomes low or undetectable and should not be used in diagnostic test clinical performance evaluation [7].

*POCT performance variability*: the significant variability observed in performance between COVID-19 tests can be explained by:

▪ Differences in test population
▪ Time of testing; proportion of early versus late COVID-19 disease stage
▪ Specimen type (nasal swab, saliva, sputum, whole blood, serum/plasma, etc.)
▪ Differences in RT-PCR protocols used as reference assay

The emerging POCTs will not necessarily meet all the criteria outlined in the WHO TPP, but the TPP will provide a framework to assist in the manufacturing of products that will meet a use case. In addition, the TPPs can be utilized to compare key features of COVID-19 POCTs and select products that best respond to the public health needs of each region. In this review, we focus on features of non-PCR COVID-19 POCTs intended for the diagnosis of acute SARS-CoV-2 infection (WHO TPP #1) and detection of prior SARS-CoV-2 infection (WHO TPP #3). Table 1 and Table 2 summarize key features of WHO and UK MHRA TPPs for antigen-/RNA- and antibody-based POCTs, respectively. We have used the WHO TPP criteria to screen and highlight top-performing COVID-19 POCTs listed on the U.S. FDA EUA [14] and the FIND SARS-CoV-2 Diagnostics Performance [15] databases (see Tables S1 and S2 in Supplementary Materials for the full list of POCTs and their features). Based on the performance data provided in the product information sheets, we chose four critical POCT characteristics to compare performance including clinical sensitivity, clinical specificity, limit of detection, and turn-around time. There are several PCR-based integrated systems that qualify as POC COVID-19 tests. These tests have been previously discussed [18] and are not the focus of this review. We primarily focused on rapid COVID-19 POCTs that can deliver results in under 30 min. We then used the WHO TPP “desirable” clinical sensitivity (≥90% for antigen/RNA and ≥95% for antibody POCTs) as a cut-off for the initial selection of top-performing rapid POCTs.

## 3. Direct POCTs to Detect Acute SARS-CoV-2 Infection

**Figure 2:**
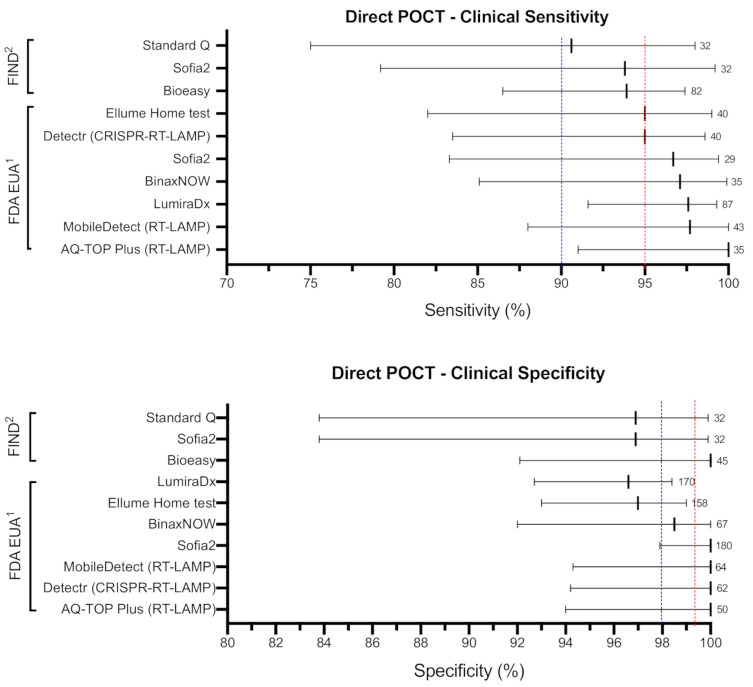
Clinical sensitivity and specificity of direct POCTs. Selected POCTs that met WHO TPP (direct detection of SARS-CoV-2) “desirable” clinical sensitivity criteria. Short vertical lines represent the calculated clinical sensitivity (top panel) and clinical specificity (bottom panel) values. Horizontal bars represent lower and upper 95% confidence intervals. Blue and red lines represent WHO TPP “acceptable” and “desirable” thresholds, respectively. Numbers represent total positive (sensitivity) and negative (specificity) clinical samples tested. All reported values were obtained from FDA, FIND, or manufacturer’s instruction for use (IFU) and have not been validated by our group. ^1^ Performance data based on manufacturer’s claims reported in FDA EUA database. ^2^ Performance data based on independent evaluation tests reported in FIND database.

**Figure 3:**
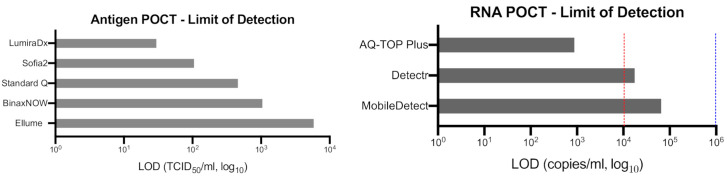
Analytical Sensitivity (limit of detection) of selected POCTs. Limit of detection (LOD) of SARS-CoV-2 antigen POCTs (**left panel**) and RNA POCTs (**right panel**), as reporter in manufacturer’s instruction for use (IFU). Blue and red lines represent WHO TPP “acceptable” and “desirable” LOD thresholds, respectively. The LOD thresholds are not available for TCID_50_/_mL_ unit.

**Figure 4:**
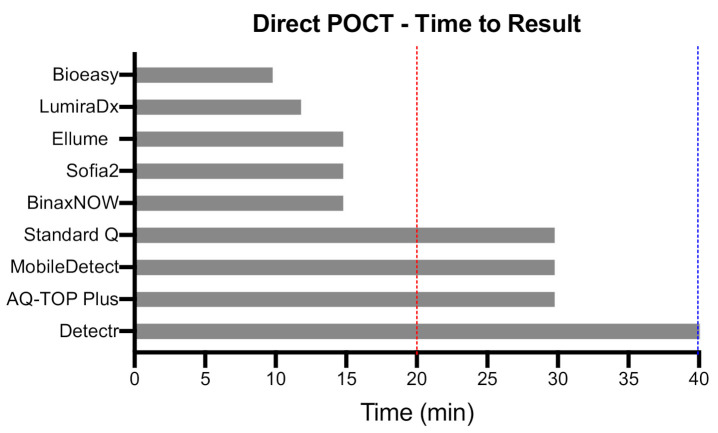
Time to result of selected direct POCTs. The time to result of direct (antigen/RNA) POCTs that met the WHO “desirable” clinical sensitivity criteria (see Figure 2) based on manufacturer’s claims. Blue and red lines represent WHO TPP “acceptable” and “desirable” thresholds, respectively.

Rapid, easy-to-use, low-cost, and relatively accurate COVID-19 POCTs can complement RT-PCR testing for timely identification of the majority of patients with early acute SARS-CoV-2 infection and avoid delays in the isolation of infected individuals. Antigen-based (e.g., lateral flow assay) and RNA-based (e.g., RT-LAMP) POCTs have the potential to detect patients with high viral loads, often during the first week of COVID-19 infection, which are most likely to transmit the virus to others [19,20]. Here, we review the clinical performance and features of several direct POCTs that meet the clinical sensitivity criteria of WHO TPP (≥90%), as shown in Table S1. We identified eight POCTs with FDA EUA and an additional three POCTs from FIND independent performance validation database that met the clinical sensitivity criteria of WHO TPP. The mean clinical sensitivity between all selected direct POCTs was 93.7 ± 7.1% with reported confidence intervals ranging from 43.7% to 100%. The mean clinical specificity among all tests was 98.8 ± 1.5% with reported confidence intervals ranging from 81.6% to 100% (Figure 2). In addition to clinical sensitivity and specificity, which can be affected by variability in specimen viral load, it is critical to assess the limit of detection (LOD) for any COVID-19 diagnostic test (see Box 2). The WHO TPP for direct POCTs sets the “acceptable” and “desirable” thresholds for LOD at 10^6^ and 10^4^ genomic copies/mL, respectively. However, due to a lack of standard LOD unit, we have reported the LOD for antigen-based tests in TCID_50_/_mL_ (overall mean ± SD of 1614.9 ± 2660 TCID_50_/_mL_) and the LOD for RNA-based tests in genomic copies/mL for (overall mean ± SD 3.2 × 10^4^ ± 3.8 × 10^4^ copies/mL) (Figure 3). The TCID_50_/_mL_ unit is defined as the viral titer concentration at which 50% of infected cells display a cytopathic effect (see Table S1). Finally, the average time to results between all tests was 23 ± 11.5 min (Figure 4).

## 4. POCTs to Detect Prior SARS-CoV-2 Infection

**Figure 5:**
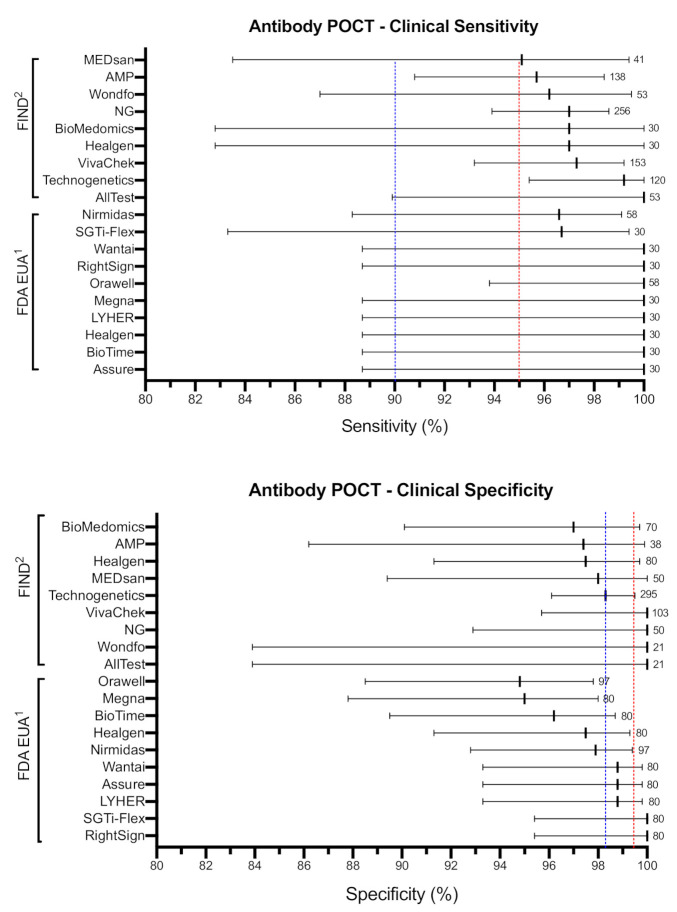
Clinical sensitivity and specificity of indirect POCTs. Selected antibody POCTs that met Table 2. infection) “desirable” clinical sensitivity cut-off. Short vertical lines represent the calculated clinical sensitivity (top panel) and clinical specificity (bottom panel) values. Horizontal bars represent lower and upper 95% confidence intervals. Blue and red lines represent the WHO TPP “acceptable” and “desirable” thresholds, respectively. Numbers represent total positive (sensitivity) and negative (specificity) clinical samples tested. All reported values were obtained from FDA, FIND, or manufacturer’s instruction for use (IFU) and have not been validated by our group. ^1^ Performance data based on manufacturer’s claims reported in FDA EUA database. ^2^ Performance data based on independent evaluation tests reported in FIND database.

**Figure 6:**
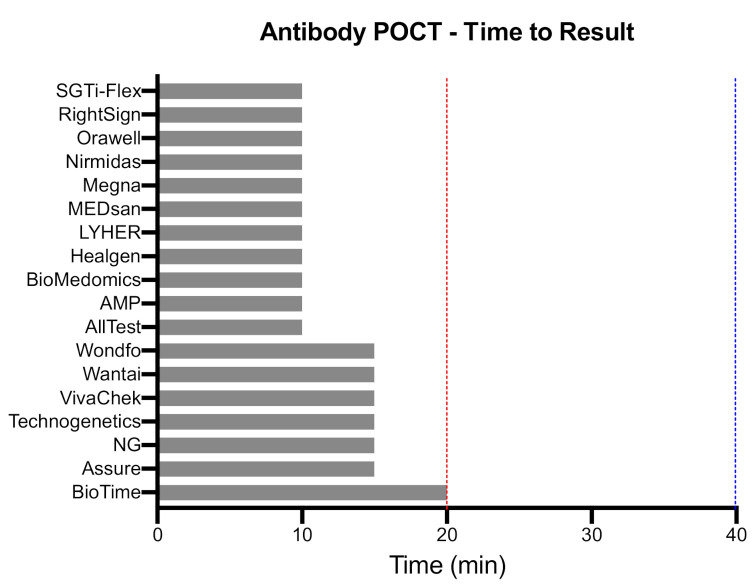
Time to result of selected indirect POCTs. The time to result of antibody POCTs that met the WHO “desirable” clinical sensitivity criteria (see Figure 2) based on manufacturer’s claims. Blue and red lines represent the WHO TPP “acceptable” and “desirable” thresholds, respectively.

SARS-CoV-2 viral load in airways becomes low or undetectable beyond two weeks post-infection, leading to diminished sensitivity of molecular tests. COVID-19 serological or antibody tests are immune-based assays (e.g., lateral flow assays) that detect immunoglobulins produced by the host in response to SARS-CoV-2 specific antigens. Studies suggest that human immunoglobulins including IgA/IgM/IgG titers remain low in the early stages of COVID-19 disease and typically become detectable (seroconversion) beyond one to two weeks after COVID-19 symptom onset, at which time, most likely, the infectiousness is decreased and some degree of immunity has developed. The IgA, IgM, or IgG antibodies reveal distinct kinetics and seroconversion timeline in response to SARS-CoV-2 that can affect the performance of antibody tests (reviewed in [8,19,21]). Therefore, antibody tests cannot play a role in clinical case management since the recency of infection cannot be determined by a single test due to substantial variability of IgA/IgM/IgG responses. However, antibody POCTs can play a complementary role to molecular tests in assessing past infections and response to SARS-CoV-2 vaccines, characterizing the dynamics of humoral response, and determining the epidemiological features of the viral outbreak including COVID-19 case fatality rates. To improve the effectiveness of antibody tests, the U.S. Centers for Disease Control and Prevention recommends to focus the testing on individuals with a high pre-test probability of having SARS-CoV-2 antibody (i.e., history of COVID-19 symptoms, contact with infected individual, or in zones with high prevalence of the virus) and the use of an orthogonal testing algorithm in which a positive test is followed up with a second test using a different antibody POCT available on the market [22]. We have recently published a detailed review of the performance of hundreds of antibody tests developed in response to the pandemic [8]. Here, we highlight the clinical performance and features of top-performing COVID-19 antibody POCTs that meet the WHO TPP “desired” sensitivity cut-off (Table 2). We identified 10 antibody POCTs with FDA EUA and an additional nine from the FIND independent performance validation database that met the clinical sensitivity criteria of WHO TPP (≥95%) (Table S2). The mean clinical sensitivity among all selected antibody POCTs was 98.3 ± 1.8%, with reported confidence intervals ranging from 83.3% to 100%. The mean clinical specificity among all tests was 98.2 ± 1.7%, with reported confidence intervals ranging from 87.0% to 100% (Figure 5). The criteria for LOD have not been defined in the WHO TPP for indirect POCTs, as currently there is no international standard to express LOD for antibody tests. The average time to results between all tests was 11.8 ± 3.0 min (Figure 6).

## 5. Concluding Remarks

The information provided by the FDA and FIND databases can be useful starting points for choosing the most appropriate COVID-19 diagnostic test to use when implementing a testing strategy. Relating critical performance characteristics to how well a product meets the relevant TPP will ensure that suboptimal tests are not used. The limitations of the use of these databases must be acknowledged, however, before finally selecting the appropriate test(s) for a specific diagnostic use case. The FDA database contains details of the data submitted by the manufacturer and reviewed for suitability by the FDA. Therefore, it is not independently confirmed data. Although the data presented from the FIND database has been generated independent of the manufacturer, it too has its limitations. There is no consensus on a standard protocol for independent validation of COVID-19 diagnostic tests. However, as noted, this information does provide an excellent starting point for considering what test to choose. Once a selection is made of potential candidates based on performance data, further considerations will be necessary before choosing the best option(s). These may include further verification of critical performance characteristics, pricing, resourcing (human and material) of the testing, quality control, as well as connectivity issues, where results can inform further public health measures, to name a few.

To date, testing for SARS-CoV-2 infection has mostly relied on RT-PCR assay. However, diagnostic technology for COVID-19 POCTs has improved at a tremendous rate in recent months. As highlighted in this review, there are several POCTs on the market that meet critical performance criteria defined by the WHO TPPs. Top-performing direct (antigen/RNA) and indirect (antibody) POCTs cited here (see Box 3) can potentially be used immediately as part of large-scale testing strategies for rapid detection of newly infected individuals or prior infections and the implementation of isolation measures. As we are months away from wide availability of vaccines, direct POCTs can play a critical role in preventing further spread of SARS-CoV-2. Additionally, antibody POCTs can be utilized to confirm and characterize vaccine-mediated immunity in a large subset of the population to ensure the efficacy and lasting effect of vaccination. Lastly, the true potential of POCTs is only realized with appropriate educational programs in place to encourage people to take advantage of available and suitable diagnostic tools and to actively participate in isolation measures designed to control the SARS-CoV-2 pandemic.

Box 3 **List of top-performing COVID-19 POCTs.**

The following COVID-19 POCTs have met the WHO TPP ‘desirable’ criteria for clinical sensitivity/specificity, LOD, and Time to Results (alphabetical order):

*Direct (antigen/RNA) POCTs*:

▪ DetectaChem MobileDetect Bio BCC19 Test (RT-LAMP)
▪ Mammoth Biosciences SARS-CoV-2 Detectr Test (RT-LAMP/CRISPR)
▪ Quidel Sofia-2 Flu+SARS Antigen Test
▪ Seasun Biomaterials AQ-TOP Plus COVID-19 Rapid Test (RT-LAMP)
▪ Shenzhen Bioeasy Biotechnology Bioeasy Diagnostic Kit COVID-19 Antigen Test

*Indirect (antibody) POCTs*:

▪ Guangzhou Wondfo Biotech Wondfo SARS-CoV-2 Ab Test (These POCTs show relatively low 95% confidence internals in sensitivity/specificity assessment)
▪ Hangzhou Biotest Biotech RightSign COVID-19 IgG/IgM Rapid Test
▪ Hangzhou Alltest Biotech AllTest COVID-19 IgG/IgM Rapid Test (These POCTs show relatively low 95% confidence internals in sensitivity/specificity assessment)
▪ NG Biotech NG IgG/IgM Rapid Test
▪ Sugentech SGTi-flex COVID-19 IgG
▪ VivaCheck Biotech COVID-19 IgM/IgG Rapid Test

## Figures and Tables

**Table 1:** Target product profiles (TPP) summary. Point-of-care rapid tests to detect SARS-CoV-2 infection.

	WHO TPP		UK MHRA TPP	
*Features*	*Acceptable*	*Desirable*	*Acceptable*	*Desirable*
Intended Use	Regions with confirmed cases, confirmed outbreaks, and in high-risk groups: early detection of SARS-CoV-2 cases where reference assays are not available or overloaded		Aid in triage of current SARS-CoV-2 infection during the acute phase of infection	Aid in triage of current SARS-CoV-2 infection at any point during active infection
Target Population	Patients with respiratory symptoms; contact with confirmed/probable COVID-19 cases; living in area of cluster		People with COVID-19 clinical signs and symptoms	People with or without COVID-19 clinical signs and symptoms
Target User/Setting	Outside laboratory in screening point of healthcare facilities by trained healthcare workers	Same but can be performed by trained lay workers	Trained healthcare worker at the point of care healthcare and non-healthcare (school, airport, prison) settings	
Target Analyte	SARS-CoV biomarker (assuming SARS-CoV-1 is not circulating)	SARS-CoV-2 only biomarker	Single SARS-CoV-2 RNA or antigen	Dual (or more) SARS-CoV-2 RNA or antigen
Sample Type	NP, OP, Nasal swab, nasal wash, sputum	Anterior nares, saliva/oral fluid, sputum	NP, OP swabs, BAL, NP, nasal wash	Sputum, saliva (not using invasive swab)
Clinical Sensitivity	≥80%	≥90%	≥80% [70–100%]	≥97% [93–100%]
Clinical Specificity	≥97%	≥99%	≥95%	≥99%
Analytical Sensitivity (LOD)	1 × 10^6^ copies/mL Ct ~ 25–30	1 × 10^4^ copies/mL Ct ~ >30	<1 × 10^4^ copies/ml	<1 × 10^2^ copies/ml
Time to Results	≤40 min	≤20 min	≤2 h	≤30 min
Result Stability	Fixed reading time	Stored image or 6 weeks	<30 min	<1 h
Storage	12 mo at 4–30 °C	18–24 mo at 4–40 °C	12 mo at 2–8 °C	12 mo at 4–30 °C

**Table 2:** TPP, point-of-care rapid tests for prior SARS-CoV-2 infection (antibody POCT).

	WHO TPP		UK MHRA TPP		
*Features*	*Acceptable*	*Desirable*	*Acceptable*		*Desirable*
Intended use	Easy to use test to detect prior SARS-CoV-2 infection		Detect prior exposure to SARS-CoV-2		Detect immunity to SARS-CoV-2
Target population	General population in survey/surveillance studies, group at high risk of exposure to SARS-CoV-2		Recovered from suspected or confirmed SARS-CoV-2 infection		Group that need to know immunity to SARS-CoV-2
Target user/setting	Outside laboratory in screening point of healthcare facilities by trained healthcare workers	Same but can be performed by trained lay workers	Health care professionals (clinics, pharmacies, workplace, non-lab settings)		Person trained in operating the test kit (clinics, pharmacies, workplace, non-lab settings)
Target analyte	At least one isotype or other biomarker specific to prior SARS-CoV-2 infection		Total antibodies to SARS-CoV-2		IgG antibodies to SARS-CoV-2
Sample type	Plasma/serum, fingerstick, saliva/oral fluids		Fingerstick blood		Fingerstick blood, venous blood, serum/plasma
Clinical sensitivity	≥90%	≥95%	≥98% (96–100%) (test min 200 positive samples)		
Clinical specificity	≥97%	≥99%	≥98% (96–100%) (test min 200 negative samples)		
Time to results	≤40 min	≤20 min	≤20 min		≤15 min
Result stability	Fixed reading time	Stored image or 6 weeks	<30 min	<1 h	
Storage	12 mo at 2–30 °C, 70% RH	18–24 mo at 2–40 °C, 90% RH	12 mo at 5–30 °C, 80% RH		

## Data Availability

Data available in a publicly accessible repository. The data presented in this study are openly available in reference number [14,15].

## Supplementary Materials

The following are available online at https://www.mdpi.com/2075-4418/11/1/115/s1, Table S1: Direct (antigen/RNA) POCTs selected based on WHO TPP criteria, Table S2: Indirect (antibody) POCTs selected based on WHO TPP criteria.
