# Supplementary material for: COVID-19 Point-of-Care Diagnostics That Satisfy Global Target Product Profiles
Source: Diagnostics (Basel). 2021 Jan 12;11(1):115. doi: 10.3390/diagnostics11010115 (PMC7828180; doi:10.3390/diagnostics11010115)
Supplement: Supplementary file 1 [file diagnostics-11-00115-s001.pdf]

Table S1: Selected direct POCT (antigen/RNA) - WHO TPP Criteria

	Wolrd Health Organization v.1.0		FDA EUA Selected Direct (antigen/RNA) POCT (performance based on manufacturer's claims)							ed Direct (antigen/RNA) POCT (performance based on independent		
	**Acceptable**	**Desirable**	**BinaxNOW COVID-19 Ag Card**	**Sofia 2 Flu+SARS Antigen FIA**	**LumiraDx SARS-CoV2 Ag Test**	**COVID-19 Home Test**	**MobileDetect Bio BCC19 Test (RT-LAMP)**	**AQ-TOP Plus COVID-19 Rapid Test (RT-LAMP)**	**SARS-CoV-2 Detectr Test (RT-LAMP)**	**Bioeasy Diagnostic kit - COVID-19 Ag test**	**Sofia 2 SARS Antigen FIA**	**Standard Q Covid-19 Ag FIA**
Manufacturer			Abbott	Quidel Corporation	LumiraDx	Ellume	DetectaChem Inc.	SEASUN BIOMATERIALS, Inc.	Mammoth Biosciences, Inc.	Shenzhen Bioeasy Biotechnology Co.,Ltd.	Quidel Corporation	SD Biosensor, INC.
Country			USA	USA	UK / USA	Australia	USA	Korea	USA	China	USA	Korea
URL			https://dam.abbott.com/en-us/homepage/coronavirus/BinaxNOW-Fact-Sheet-Final.pdf	https://www.quidel.com/immunoassays/sofia-2-flu-sars-antigen-fia	https://www.lumiradx.com/us-en/what-we-do/diagnostics/test-technology/antigen-test	https://www.ellumehealth.com	https://mobiledetectbio.com/	http://www.seasunbio.com/english/products4.html	https://mammoth.bio/covid/	http://en.bioeasy.com/?p=1038	https://www.quidel.com/immunoassays/sofia-2-flu-sars-antigen-fia	http://sdbiosensor.com/xe/product/7672
**Intended Use**	1) In areas with confirmed SARS-CoV-2 community wide transmission or confirmed outbreaks in closed or semi-closed communities and in high risk groups; 2) In suspected SARS-CoV-2 outbreak situations; 3) Monitor trends in disease incidence		qualitative detection of SARS-CoV-2 antigen in patient care setting; POCT	qualitative detection of SARS-CoV-2 antigen in patient care setting; POCT	qualitative detection of SARS-CoV-2 antigen in patient care setting; POCT	qualitative detection of SARS-CoV-2 antigen; non-prescription home use	qualitative detection of SARS-CoV-2 RNA	qualitative detection of SARS-CoV-2 RNA	qualitative detection of SARS-CoV-2 RNA	qualitative detection of SARS-CoV-2 in patient care setting; POCT	qualitative detection of SARS-CoV-2 in patient care setting; POCT	qualitative detection of SARS-CoV-2 in patient care setting; POCT
**Target Population / patient**	patients with respiratory symptoms or fever or other symptoms (diarrhoea, anosmia) and either a known contact with a confirmed or probable COVID-19 patient OR living in an area of cluster OR community transmission, and close contact of index patients (confirmed COVID-19 patients)		individuals suspected of COVID-19 within 7 days of sympton onset	individuals suspected of COVID-19 within 5 days of sympton onset	individuals suspected of COVID-19 within 12 days of sympton onset	individuals suspected of COVID-19	individual suspected of COVID-19	individual suspected of COVID-19	individual suspected of COVID-19	individual suspected of COVID-19	individuals suspected of COVID-19 within 5 days of sympton onset	post
**Target use setting**	outside laboratories including routien screening points of health care facilities by health care workers or lab techs with appropriate training to use the test.	same as acceptable but can be self-administered and/or performed by trained lay workers (volunteer, community health workers)	patient care setting; CLIA certified labs	patient care setting; CLIA certified labs	patient care setting; CLIA certified labs	non-prescription home use	certified laboratories	certified laboratories	certified laboratories	not specified (patient care setting / labs)	patient care setting; CLIA certified labs	not specified (patient care setting / labs)
**Target molecule**	SARS-CoV biomarker (RNA, antigen) specific for acute (first week after onset of symptoms/current infection)	SARS-CoV-2 only biomarker specific for acute and subacute (first 2 weeks after onset of symptoms/current infection)	SARS-CoV-2 nucleocapsid protein (and SARS-CoV)	SARS-CoV-2 nucleocapsid protein	SARS-CoV-2 nucleocapsid protein	SARS-CoV-2 & SARS-CoV nucleocapsid protein	SARS-CoV-2 N and E genes	SARS-CoV-2 ORF1ab and N genes	SARS-CoV-2 N genes	n/a	SARS-CoV-2 nucleocapsid protein	SARS-CoV-2 nucleocapsid protein
**Analytical sensitivity / LOD**	~ 10^6 genomic copies/ml	~ 10^4 genomic copies/ml	22.5 $TCID_{50}$/swab (1125 TCID50/ml)	113 $TCID_{50}$/ml	32 $TCID_{50}$/ml	6310 $TCID_{50}$/ml	75 copies/µl	1 copies/µl	20 copies/µl	n/a	113 TCID50/ml	494.5 TCID50/ml
**Analytical specificity**	detects all SARS-CoV-2 viral strains and dose not cross react with other human coronaviruses (except SARS-CoV-1) or any other common diseases with similar symptoms (influenza A, B, RSV, malaria, dengue)	detects all SARS-CoV-2 virla strains and dose not cross react with other human CoV or common diseases with similar symptoms	no cross-reactivity in a panel of high prevalence respiratory pathogens	no cross-reactivity in a panel of high prevalence respiratory pathogens	no cross-reactivity in a panel of high prevalence respiratory pathogens	no cross-reactivity in a panel of high prevalence respiratory pathogens	no cross-reactivity in a panel of high prevalence respiratory pathogens	no cross-reactivity in a panel of high prevalence respiratory pathogens	no cross-reactivity in a panel of high prevalence respiratory pathogens	n/a	no cross-reactivity in a panel of high prevalence respiratory pathogens	n/a
**Sensitivity (or PPA)**	≥ 80%	≥ 90%	97.1% (85.1 - 99.9)	96.7((83.3-99.4)	97.6% (91.6-99.3)	95% (82-99)	97.7% (88-100)	100% (91-100)	95% (83.5.98.6)	93.9% (86.5-97.4)	93.8% (79.2-99.2)	90.6% (75-98)
**Specificity (or NPA)**	≥ 97%	≥ 99%	98.5% (92.0-100%)	100% (97.9-100)	96.6% (92.7-98.4)	97% (93-99)	100% (94.3-100)	100% (94-100)	100% (94.2-100)	100% (92.1-100)	96.9% (83.8-99.9)	96.9% (83.8-99.9)
**Type of analysis**	qualitative (yes/no), semi-quantitative, or quantitative	N/A	lateral flow immunoassay	lateral flow immunofluorescent	microfluidic immunofluorescence	lateral flow immunofluorescent	RT-LAMP	RT-LAMP	CRISPR-based RT-LAMP	fluorescent immunochromatographic, double antibody sandwich rxn	lateral flow immunofluorescent	fluorescent immunoassay
**Interpretation**	visual manual and/or hardware reader (proprietary or smart phone)	visual manual read or digital readout via smartphone app reader with connectivity	visual manual read	digital readout	digital readout	digital readout (smartphone)	visual color recognition	digital readout	digital readout	digital reader, fluorescence analyzer	digital readout	digital readout
**Sample type**	nasopharyngeal, oropharyngeal, nasal swab, nasal wash, sputum	anterior nares (swab), saliva/oral fluid, sputum	nasal swab	nasal swab	nasal swab	nasal swab	nasal swab	nasal swab; BAL	nasal swab / wash	nasal swab	nasal swab	nasal swab
**Sample collection device**	compatible with an existing swab material	compatible with multiple swab materials (self-collection or no swab e.g. saliva)	swabs in the kit ONLY	swabs in the kit ONLY	recommended swab (not included)	swabs in the kit ONLY	recommended swab (not included)	recommended swab (not included)	recommended swab (not included)	not provided	swabs in the kit ONLY	swab provided in kit
**End use profile**	trained staff in health care facilities	trained staff or community level (lay person) or self-administered	medical professional or trained staff in certified patient care setting	trained clinical laboratory personnel and individuals trained in point of care settings	trained clinical laboratory personnel and individuals trained in point of care settings	home use	trained lab personnel	trained lab personnel	trained lab personnel	trained medical / lab operator	trained clinical laboratory personnel and individuals trained in point of care settings	professional use
**Training needs**	0.5 days with instructions for use and quick reference guide(s)	2hr with instructions, quick reference guide including smart phone app(s)	N/A (procedure card included)	N/A	N/A	no	Yes	Yes	Yes	n/a	N/A	n/a
**Sample preparation steps**	1	0	1	1	1	1	1	1	1	1	1	1
**Reagents reconsitituion**	very simple reconstituion	ready to use	ready to use	ready to use	ready to use	ready to use	ready to use	ready to use	ready to use	ready to use	ready to use	ready to use
**Sample min volume**	single swab and minimal extraction buffer/diluent		single swab + extraction buffer	single swab + extraction buffer	single swab + extraction buffer	single swab + extraction buffer	single swab + extraction buffer	single swab + extraction buffer	single swab + extraction buffer	single swab + extraction buffer	single swab + extraction buffer	single swab + extraction buffer
**Need to transfer a precise volume of sample**	autofill or graduated volume markings on sample transfer device	no, or limited to a number of drops	no, few drops	no, few drops	no, few drops	no, few drops	pipette fixed volumes	pipette fixed volumes	pipette fixed volumes	no, few drops	no, few drops	no, few drops
**Number of timed steps**	≤ 3	1, with the potential for digitally guided workflows and built-in timers to reduce user errors on timed steps	1	1	1	1	2	2	2	1 (automatic by analyzer)	1	1
**Time to result**	≤ 40 min	≤ 20 min	15min	15min	12min	15min	30min	30min	45min	10min	15min	30min
**Sample stability pre-testing**	30min (10-35oC); 2-4hr (4-8oC); several days frozen (min -20oC)	test compatible with both dry and preserved samples; 3hr (10-40oC); 24hr (4-8oC); months frozen	<1hr room temp	48hr room temperature or 2-8oC	5hr room temp; 5 days frozen -80oC	n/a	n/a	n/a	n/a	<24hr at 2-8OC; or -70oC long-term	48hr room temperature or 2-8oC	24h room temperature; 2-8oC for 48hr
**Result validity stability**	fixed reading time	stored image or 6 weeks	<30min	<30min	n/a	n/a	n/a	n/a	n/a	n/a	<30min	n/a
**Invalid rate**	<2% invalid results with correct use by operator	<0.5% invalid results with correct use by operator	n/a	n/a	n/a	8%	n/a	n/a	n/a	n/a	n/a	n/a
**Operating conditions**	15-35oC, 25-80% RH up to 1500m	10-40oC; 25-90% RH up to 3000m	Room temperature	room temperature	10-30 oC; RH 10-90%	room temperature	room temperature	room temperature	room temperature	2-35oC; RH 40-90%	room temperature	room temperature
**Test kit stability and storage**	12mo 4-30oC; tolerates brief period >40oC; RH 75%	18-24mo at 4-40oC; tolerates freezing and brief period >40oC; RH 75%	2-30oC; expiration n/a	room temperature, 15-30oC	2-8oC	2-30oC	freeze at -20oC	freeze at -20oC	freeze at -20oC	2-30 oC	room temperature, 15-30oC	room temperture (2-30oC)
**Stability of kit once opened**	30min	1hr	n/a (use immediately)	12hr	30 day	60min	n/a	n/a	n/a	60min	12hr	n/a
**Specimen capacity & throughput**	>5/hr per operator	>10/hr per operator	n/a	n/a	n/a	n/a	n/a	n/a	1500 sample in 8hr	n/a	n/a	n/a
**Safety precautions**	standard respiratory sample collection safety precautions recommended;	tests that minimize the need for biosafety requirement are strongly preferred (e.g. with a self-sample collection device with virus inactivation)	standard respiratory sample precautions	standard respiratory sample precautions	standard respiratory sample precautions	standard respiratory sample precautions	standard respiratory sample precautions	standard respiratory sample precautions	standard respiratory sample precautions	standard respiratory sample precautions	standard respiratory sample precautions	standard respiratory sample precautions
**Quality control**	internal control (for sample flow/migration); positive and negative controls sold separately;	internal control; lyophilized positive control and negative control provided in the kit;	internal control; pos/neg control	internal control; pos/neg control	internal control; pos/neg control	internal control	internal control; pos/neg control	internal control; pos/neg control	internal control; pos/neg control	internal control	internal control; pos/neg control	internal control; pos/neg controls separate
**Remote connectivity capacity**	not required for reader independent tests; if device-based: remote export of data possible	test is compatible with readers and other data capture devices; internal memory to store results even if power cut and with the ability to report to country health information management systems using on onboard unique identifier or other personal data protection safetguard linking the test to the user.	no	n/a	optional	Smartphone compatible	n/a	n/a	n/a	n/a	n/a	n/a
**Need for additional equipment**	handheld or on desktop (<1kg); battery or solar power operated; >8hr rechargable battery life	no additional equipment required, but if required, it should have potential for digital connectivity through smartphone applications	no	Sofia / Sofia 2 instrument	LumiraDx Instrument (portable)	Smartphone	MD-Bio Heater (or thermal cycler)	CFX 96 PCR system	CFX 96 PCR system	Yes, fluorescece analyzer	Sofia / Sofia 2 instrument	Standard F Analyzer
**Need for maintenance/spare parts**	none, swap out or replace ancillary device when needed	none required as device-free	n/a	n/a	n/a	n/a	n/a	n/a	n/a	none required	n/a	n/a
**Waste/disposal requirements**	routine biohazard waste	not applicable	biohazard waste	biohazard waste	biohazard waste	General waste	biohazard waste	biohazard waste	biohazard waste	biohazard waste	biohazard waste	biohazard waste
**Manufacturing**	ISO 13485: 2016 compliant	WHO or stringent regulatory authority emergency use listing/authorization or WHO prequalification or other stringent regulatory authority approval	FDA EUA	FDA EUA	FDA EUA	FDA EUA	FDA EUA	FDA EUA	FDA EUA	CE	FDA EUA	CE
**Accessibility**	to maximize accessiblity, all product manufacturers should have the capability to rapidly scale-up production and offer the product at a cost that allows borad use, including in low- and middle-income countries.		n/a	n/a	n/a	n/a	n/a	n/a	n/a	n/a	n/a	n/a

Table S2: Selected indirect POCT (antibody) - WHO TPP Criteria

	Wolrd Health Organization v.1.0		FDA EUA Selected Antibody POCT				
	Acceptable	Desirable	COVID-19 IgG/IgM Rapid Test	Wantai SARS-CoV-2 Ab Rapid Test	RightSign COVID-19 IgG/IgM Rapid Test	LYHER COVID-19 IgM/IgG Combo Test	healgen COVID-19 IgG/IgM Rapid Test
Manufacturer			Assure Tech. (Hangzhou) Co. Ltd.	Beijing Wantai Biological Pharmacy Enterprise Co., Ltd.	Hangzhou Biotest Biotech Co., Ltd.	Hangzhou Laihe Biotech Co., Ltd	Verify Diagnostics
Country			China	China	China	China	USA
URL			http://www.diareagent.com/contact.html	https://www.fda.gov/media/140030/download	https://www.fda.gov/media/138657/download	https://www.fda.gov/media/139409/download, http://www.lyher.com/default.aspx?pageid=14&pageType=detail&id=54	https://verifydiagnostics.com/healgen-covid-19-igg-igm-rapid-test-receives-fda-eua-approval/
**Intended Use**	easy to use test that can be used at the POC to detect prior SARS-CoV-2 infection		POC in patient care setting; Labs; not home use	professional use only; not home use	professional use only; not home use	professional use only; not home use	professional use only; not home use
**Target Population / patient**	General population invovled in surveys; groups at high risk of exposure to		general population	general population	general population	general population	general population
**Target use setting**	outside labs; health facilities; community settings; by health care workers or lab techs	same as acceptable + can be performed by trained lay workers	trained lab personnel; not home use.	trained lab personnel; not home use.	trained lab personnel; not home use.	trained lab personnel; not home use.	trained lab personnel; not home use.
**Target molecule**	at least one isotype; other biomarkers specific to prior infection	n/a	NP, Spike	Spike	spike RBD domain	S1 spike domain	S1 spike domain
**Analytical sensitivity / LOD**	currently no standard/units to express LOD; min detectable concentration of analyte	n/a	n/a	n/a	n/a	n/a	n/a
**Analytical specificity**	detect only SARS-CoV-2 specific isotype or biomarker	n/a	n/a	n/a	n/a	n/a	n/a
**Sensitivity (PPA)**	>90%	>95%	100% (88.7-100)	100% (88.7-100)	100% (88.7-100)	100% (88.7-100)	100% (88.7-100)
**Specificity (NPA)**	>97%	>99%	98.8% (93.3-99.8)	98.8% (93.3-99.8)	100% (95.4-100)	98.8% (93.3-99.8)	97.5% (91.3-99.3)
**Type of analysis**	qualitative	n/a	qualitative; lateral flow	qualitative; lateral flow	qualitative; lateral flow	qualitative; lateral flow	qualitative; lateral flow
**Interpretation**	visual; portable automated reader	visual - device free	visual	visual	visual	visual	visual
**Sample type**	plasma; serum;	n/a	whole blood; serum/plasma; fingerstick blood	whole blood; serum/plasma; fingerstick blood	whole blood; serum/plasma	serum/plasma	whole blood; serum/plasma; not fingerstick blood
**Test kit format**	kit contains material for sample collection	same plus controls	intenal control; pos/neg control not included	intenal control; pos/neg control not included	intenal control; pos/neg control not included	intenal control; pos/neg control not included	intenal control; pos/neg control not included
**End use profile**	Health care facility worker	Lay person / non-professional staff	trained lab personnel	trained lab personnel	trained lab personnel	trained lab personnel	trained lab personnel
**Training needs**	<0.5 days	<2hr (quick reference)	n/a	n/a	n/a	n/a	n/a
**Sample preparation steps**	1	0	0	0	0	1 (separate plasma/serum)	0
**Reagents reconsitituion**	if very simple to do	no, ready to use	ready to use	ready to use	ready to use	ready to use	ready to use
**Need to transfer a precise volume of sample**	if autofill or volume marking	no or a few drops	fill line	10ul	fill line (10ul)	10ul	fill line (5ul)
**Number of timed steps**	<2	1	1	1	1	1	1
**Time to result**	<40min	<20min	15min	15min	10min	10min	10min
**Sample stability pre-testing**	30min RT (10-35oC); 8hr (2-8oC)	3hr RT (10-35oC); 24hr (2-8oC)	whole blood 3 days at 2-8oC; serum/plasma 7 days 2-8oC	serum/plasma 7 day 2-8oC or 3 wks -15oC; whole blood used immediately	serum/plasma 3 days at 2-8oC; -20oC for longer-term; whole blood 2 days at 2-8oC; do not freeze blood	2-8oC for 2 days; <-20oC long-term	serum/plasma 3 days at 2-8oC; -20oC for 1mo; whole blood 2 days at 2-8oC; do not freeze blood
**Result validity stability**	fixed reading time	stored images or 6 weeks	30min	20min	20min	15min	15min
**Invalid rate**	<5% invalid results with correct use	<1% invalid results with correct use	n/a	n/a	n/a	n/a	n/a
**Operating conditions**	15-35oC; 20-80% RH; altitude 1500m	10-40oC; 25-90% RH; altitude 3000m	room temperature	room temperature	room temperature	room temperature	room temperature
**Test kit stability and storage**	12mo, 2-30oC, 70% RH;	18-24mo, 2-40oC, 90% RH	2-30oC;	2-30oC for 9mo	2-30oC;	2-30oC, dark	2-30oC;
**Stability of kit once opened**	1hr	4hr	1hr	0.5hr	1hr	0.5hr	1hr
**Specimen capacity & throughput**	>5/hr per operator	>10/hr per operator	n/a	n/a	n/a	n/a	n/a
**Safety precautions**	standard blood collection safety precautions	n/a	standard	standard	standard	standard	standard
**Quality control**	internal control (sample flow/migration); positive / negative controls sold separately	internal control; lyophilized positive/negative conrols provided	internal control; positive, negative controls optional	internal control; positive, negative controls optional	internal control; positive, negative controls optional	internal control; positive, negative controls optional	internal control; positive, negative controls optional
**Remote connectivity capacity**	device-based: remote export of data possible; no device: export through separate 3rd party reader	test is compatible with readers and other data capture devices	no	no	no	no	no
**Need for additional equipment**	handheld: <1kg; battery or solar power; >6hr rechargable battery life	no additional equipment	no	no	no	no	no
**Need for maintenance/spare parts**	none, swap out or replace device	none required as device free	no	no	no	no	no
**Waste/disposal requirements**	standard biohazardous waste disposal	smal environmental footprint; recyclable or compostable plastic	biohazard waste	biohazard waste	biohazard waste	biohazard waste	biohazard waste
**Accessibility**	to maximize accessibility, all product manufacturers should have the capability to rapidly scale-up production and offer the product at a cost that allows broad use, including in low- and middle-income countries.		n/a	n/a	n/a	n/a	n/a

	FDA EUA Selected Antibody POCT				
	Orawell IgM/IgG Rapid Test	Rapid COVID-19 IgM/IgG Combo Test	COVID-19 IgM/IgG Antibody Detection Kit	SGTi-flex COVID-19 IgG	SARS-CoV-2 IgG/IgM Rapid Test
Manufacturer	Jiangsu Well Biotech Co., Ltd.	Megna Health Inc.	Nirmidas Biotech	Sugentech, Inc.	Xiamen Biotime Biotechnology Co., Ltd.
Country	China	USA	USA	Korea	China
URL	https://www.fda.gov/media/142422/download	https://www.megnahealth.com/products/#rapid-antibody-test	https://www.nirmidas.com/nirmidas-covid19-igmigg-antibody-detection-kit-eua-authorized	https://sugentech.com/products/products-view.php?ct=7&target=32%27	https://www.fda.gov/media/140443/download
**Intended Use**	professional use only; not home use	professional use only; not home use	professional use only; not home use	professional use only; not home use	professional use only; not home use
**Target Population / patient**	general population	general population	general population	general population	general population
**Target use setting**	trained lab personnel; not home use.	trained lab personnel; not home use.	trained lab personnel; not home use.	trained lab personnel; not home use.	trained lab personnel; not home use.
**Target molecule**	spike RBD domain	NP	S1 & RBD domains of Spike	NP & Spike RBD	spike
**Analytical sensitivity / LOD**	n/a	n/a	n/a	n/a	n/a
**Analytical specificity**	n/a	n/a	n/a	n/a	n/a
**Sensitivity (PPA)**	100% (93.8-100)	100% (88.7-100)	96.6% (88.3-99.1)	96.7% (83.3-99.4)	100% (88.7-100)
**Specificity (NPA)**	94.8% (88.5-97.8)	95% (87.8-98)	97.9% (92.8-99.4)	100% (95.4-100)	96.2% (89.5-98.7)
**Type of analysis**	qualitative; lateral flow	qualitative; lateral flow	qualitative; lateral flow	qualitative; lateral flow	qualitative; lateral flow
**Interpretation**	visual	visual	visual	visual	visual
**Sample type**	whole blood; serum/plasma; not fingerstick blood	serum/plasma	serum/plasma	venous blood, serum, plasma (not figerstick)	venous blood, serum, plasma (not figerstick)
**Test kit format**	intenal control; pos/neg control not included	intenal control; pos/neg control not included	intenal control; pos/neg control not included	intenal control; pos/neg control not included	intenal control; pos/neg control not included
**End use profile**	trained lab personnel	trained lab personnel	trained lab personnel	trained lab personnel	trained lab personnel
**Training needs**	n/a	n/a	n/a	n/a	n/a
**Sample preparation steps**	0	1 (separate plasma/serum)	1 (separate plasma/serum)	0	0
**Reagents reconsitituion**	ready to use	ready to use	ready to use	ready to use	ready to use
**Need to transfer a precise volume of sample**	fill line (10ul)	fill line (10ul)	fill line (10ul)	10ul	one drop (10-15ul)
**Number of timed steps**	1	1	1	1	1
**Time to result**	10min	10min	10min	10min	20min
**Sample stability pre-testing**	serum/plasma 2-8oC for 5 days; -70oC for longterm;	serum/plasma <3 days at 2-8oC; -20oC for <3mo; -70oC longterm;	4-8oC <3 days; -20oC <1 mo;	2-8oC <5 days; -70oC longterm;	serum/plasma <14 days at 2-8oC; -20oC for <3mo; whole blood 2-8oC <3 days;
**Result validity stability**	15min	20min	15min	30min	30min
**Invalid rate**	n/a	n/a	n/a	n/a	n/a
**Operating conditions**	room temperature	room temperature	room temperature	room temperature	room temperature
**Test kit stability and storage**	2-8oC <9mo;	4-30oC;	4-25oC	2-8oC	5mo at RT; 9mo at 2-8oC
**Stability of kit once opened**	2-8oC <7 days	use immediately	15min	30 days at 2-8oC	use immediately
**Specimen capacity & throughput**	n/a	n/a	n/a	n/a	n/a
**Safety precautions**	standard	standard	standard	standard	standard
**Quality control**	internal control; positive, negative controls optional	internal control; positive, negative controls optional	internal control; positive, negative controls optional	internal control; positive, negative controls optional	internal control; positive, negative controls optional
**Remote connectivity capacity**	no	no	no	no	no
**Need for additional equipment**	no	no	no	no	no
**Need for maintenance/spare parts**	no	no	no	no	no
**Waste/disposal requirements**	biohazard waste	biohazard waste	biohazard waste	biohazard waste	biohazard waste
**Accessibility**	n/a	n/a	n/a	n/a	n/a

	FIND Selected Antibody POCT								
	COVID-19 IgM/IgG Gold	AllTest COVID-19 IgG/IgM Rapid Test	COVID-19 IgM/IgG Rapid Test	Healgen COVID-19 IgM/IgG Rapid Test	COVID-19 IgM/IgG Rapid Test	Wondfo SARS-CoV-2 Ab Test	AMP Rapid SARS-CoV-2 IgM/IgG Test	SARS-CoV-2 IgM/IgG Rapid Test	NG IgG/IgM Rapid Test
Manufacturer	Technogenetics srl	Hangzhou Alltest Biotech Co., Ltd.	VivaChek Biotech (Hangzhou) Co., Ltd.	Verify Diagnostics	BioMedomics, Inc.	Guangzhou Wondfo Biotech Co., Ltd., China	AMEDA Labordiagnostik GmbH	MEDsan GmbH	NG Biotech Laboratoires
Country	Italy	China	China	USA	USA	China	Austria	Germany	France
URL	https://www.technogenetics.it/it/prodotti/covid-19/rapid-test/rapid-test-sars-cov-2-igm-igg-gold	http://www.alltests.com.cn/EN/ProductMain.asp?cclassid=2&classid=51	https://www.vivachek.com/en/prods/prod-rapidtest.html	https://verifydiagnostics.com/healgen-covid-19-igg-igm-rapid-test-receives-fda-eua-approval/	https://www.biomedomics.com/products/infectious-disease/covid-19-rt/	https://www.bilcare.com/SARS-CoV-2%20Antibody%20Test%20(Lateral%20Flow%20Method).pdf	https://www.amp-med.com/news-rapid-test-sars-cov-2	https://www.medsan.eu/en/detection/	https://ngbiotech.com/2020/07/13/ng-test-igg-igm-covid-19-obtains-trod-status/
**Intended Use**		for professionals only; not home use	for professionals only; not home use	for professionals only; not home use	for professionals only; not home use	n/a	for professionals only; not home use	for professionals only; not home use	for professionals only; not home use
**Target Population / patient**		general population	general population	general population	general population	general population	general population	general population	general population
**Target use setting**		trained lab personnel; not home use.	trained lab personnel; not home use.	trained lab personnel; not home use.	trained lab personnel; not home use.	n/a	trained lab personnel; not home use.	trained lab personnel; not home use.	trained lab personnel; not home use.
**Target molecule**		n/a	n/a	S1 spike domain	n/a	n/a	n/a	n/a	n/a
**Analytical sensitivity / LOD**		n/a	n/a	n/a	n/a	n/a	n/a	n/a	n/a
**Analytical specificity**		n/a	n/a	n/a	n/a	n/a	n/a	n/a	n/a
**Sensitivity (PPA)**	99.2% (95.4-100)	98.1% (89.9-100)	97.30%	97% (82.8-100)	97% (82.8-100)	96.2% (87-99.5)	95.7% (90.8-98.4)	95.1% (83.5-99.4)	96.9% (93.9-98.6)
**Specificity (NPA)**	98.3% (96.1-99.5)	100% (83.9-100)	100%	97.5% (91.3-99.7)	97.1% (90.1-99.7)	100% (83.9-100)	97.4% (86.2-99.9)	98% (89.4-100)	100% (92.9-100)
**Type of analysis**		qualitative; lateral flow	qualitative; lateral flow	qualitative; lateral flow	qualitative; lateral flow	qualitative; lateral flow	qualitative; lateral flow	qualitative; lateral flow	qualitative; lateral flow
**Interpretation**		visual	visual	visual	visual	visual	visual	visual	visual
**Sample type**		whole blood (fingertip/venous), serum, plasma	whole blood (fingertip/venous), serum, plasma	whole blood; serum/plasma; not fingerstick blood	whole blood (capilary, venous), serum, plasma	whole blood/serum/plasma	whole blood/serum/plasma	whole blood (fingerstick), serum, plasma	whole blood (fingerstick), serum, plasma
**Test kit format**		intenal control; pos/neg control not included	n/a	intenal control; pos/neg control not included	intenal control; pos/neg control not included	n/a	n/a	intenal control; pos/neg control not included	intenal control; pos/neg control not included
**End use profile**		trained lab personnel	trained lab personnel	trained lab personnel	trained lab personnel	trained lab personnel	trained lab personnel	trained lab personnel	trained lab personnel
**Training needs**			n/a	n/a	n/a	n/a	n/a	n/a	n/a
**Sample preparation steps**		0	0	0	0	n/a	0	0	0
**Reagents reconsitituion**		ready to use	ready to use	ready to use	ready to use	n/a	ready to use	ready to use	ready to use
**Need to transfer a precise volume of sample**		10ul (fill line)	10ul (fill line)	fill line (5ul)	15ul blood; 10ul serum/plasma	n/a	10ul whole blood, 5ul serum/plasma	10ul (fill line)	n/a
**Number of timed steps**		1	1	1	1	n/a	n/a	1	n/a
**Time to result**		10min	15min	10min	10min	15min	10min	10min	15min
**Sample stability pre-testing**		serum/plasma 7 days at 2-8oC; -20oC for long-term; whole blood 2 days at 2-8oC; do not freeze blood	n/a	serum/plasma 3 days at 2-8oC; -20oC for 1mo; whole blood 2 days at 2-8oC; do not freeze blood	anticoagulated blood 2-8oC for 5 days; serum/plasma 208oC 5 days or -20oC longterm;	n/a	n/a	serum/plasma 3 days at 2-8oC; -20oC for longterm; whole blood 2 days at 2-8oC; do not freeze blood	n/a
**Result validity stability**		20min	20min	15min	15min	n/a	15min	15min	n/a
**Invalid rate**		n/a	n/a	n/a	n/a	n/a	n/a	n/a	n/a
**Operating conditions**		room temperature	room temperature	room temperature	room temperature	room temperature	room temperature	room temperature	room temperature
**Test kit stability and storage**		2-30oC	2-30oC	2-30oC;	2-30oC;	2-30oC;	2-30oC;	2-30oC;	2-30oC;
**Stability of kit once opened**		n/a	n/a	1hr	1hr	n/a	n/a	n/a	n/a
**Specimen capacity & throughput**		n/a	n/a	n/a	n/a	n/a	n/a	n/a	n/a
**Safety precautions**		standard	standard	standard	standard	n/a	standard	standard	n/a
**Quality control**		n/a	n/a	internal control; positive, negative controls optional	internal control; positive, negative controls optional	n/a	internal control; positive, negative controls optional	internal control; positive, negative controls optional	n/a
**Remote connectivity capacity**		no	no	no	no	n/a	n/a	n/a	n/a
**Need for additional equipment**		no	no	no	no	n/a	n/a	n/a	n/a
**Need for maintenance/spare parts**		no	no	no	no	n/a	n/a	n/a	n/a
**Waste/disposal requirements**		biohazard waste	biohazard waste	biohazard waste	biohazard waste	n/a	biohazard waste	biohazard waste	n/a
**Accessibility**		n/a	n/a	n/a	n/a	n/a	n/a	n/a	n/a
